# Making Memories Matter

**DOI:** 10.3389/fnint.2012.00116

**Published:** 2012-12-18

**Authors:** Paul E. Gold, Donna L. Korol

**Affiliations:** ^1^Department of Biology, Syracuse UniversitySyracuse, NY, USA

**Keywords:** epinephrine, glucose, arousal, emotion, memory, learning strategy

## Abstract

This article reviews some of the neuroendocrine bases by which emotional events regulate brain mechanisms of learning and memory. In laboratory rodents, there is extensive evidence that epinephrine influences memory processing through an inverted-U relationship, at which moderate levels enhance and high levels impair memory. These effects are, in large part, mediated by increases in blood glucose levels subsequent to epinephrine release, which then provide support for the brain processes engaged by learning and memory. These brain processes include augmentation of neurotransmitter release and of energy metabolism, the latter apparently including a key role for astrocytic glycogen. In addition to up- and down-regulation of learning and memory in general, physiological concomitants of emotion and arousal can also switch the neural system that controls learning at a particular time, at once improving some attributes of learning and impairing others in a manner that results in a change in the strategy used to solve a problem.

## Introduction

Hormonal responses to an emotional experience regulate memory for that experience (e.g., Gold and McGaugh, 1975; Gold, 1992; Cahill and McGaugh, 1998; Korol and Gold, 2007, 2008; de Quervain et al., 2009; Gold and Korol, 2010; Schwabe et al., 2012; Campolongo and Roozendaal, 2011; Sandi, 2011). The hormonal regulators of memory include adrenal, gonadal, and stress steroids as well as adrenal catecholamines. Of these, glucocorticoids and epinephrine respond acutely to the emotional context of an experience and appear to regulate both the strength and quality of emotional memories. Glucocorticoids have received the most attention in this respect, as noted by several recent reviews of the steroid’s effects on memory (e.g., Campolongo and Roozendaal, 2011; Schwabe et al., 2010, 2012; Sandi, 2011). Of note, the effects of glucocorticoids and epinephrine on memory appear to have several points of convergence. In particular, regulation of memory by these hormones is blocked by β-adrenergic receptor antagonists injected either centrally (Quirarte et al., 1997; Clayton and Williams, 2000; Roozendaal et al., 2006; Wichmann et al., 2012) or peripherally (Gold and van Buskirk, 1978; Parfitt et al., 2012). Although generally attributed to central actions, peripheral effects of adrenergic blockade are likely to interfere with peripheral actions of epinephrine, including the subsequent breakdown of hepatic glycogen stores and liberation of glucose into the blood. Considerable evidence by us and others supports the view that peripheral endocrine events are key modulators of memory.

We discuss here evidence showing that one consequence of an emotional experience, the release of epinephrine from the adrenal gland, is a particularly important memory-enhancing process. Epinephrine effects on memory are mediated, at least in part, by subsequent increases in blood glucose levels. Glucose, in turn, can enhance memory by direct actions on the brain, and likely does so by modulating glia as well as neurons. Moreover, epinephrine enhances the durability of plasticity in a synaptic model of memory, termed long-term potentiation (LTP). These enhancing actions of epinephrine and glucose reflect acute actions that are temporally associated with the time of learning. However, under conditions of high circulating levels, e.g., after high stress or high injection dose, glucose and epinephrine can impair memory, providing a physiological substrate for the classic Yerkes-Dodson inverted-U relationship between arousal and learning and memory (Yerkes and Dodson, 1908).

In addition to providing a mechanism by which high emotion results in more robust memory for the event that initiated the emotion, neuroendocrine responses to experience can also shift the type of information or an experience’s attribute to be remembered. These findings stem from studies showing that stress and arousal can alter the relative participation of multiple memory systems in a way that alters the strategy employed to solve a problem. This action is one shared by other hormones, particularly estrogens, and leads at once to better learning on some tasks and poorer learning on others. In contrast to the actions of epinephrine and glucose described above, the slower effects of estrogens and glucocorticoids, may be slower in action, setting a platform on which memories are formed (Korol and Gold, 2007; Schwabe et al., 2010).

These neuroendocrine events now known to modulate learning and memory result in conditions in which memory can be enhanced or impaired, but can also result in both enhancement and impairment at once depending on the cognitive attributes and brain regions engaged during learning. This review will describe evidence for these multiple and sometimes opposing cognitive effects of hormonal concomitants of emotion, primarily considering results obtained in laboratory rodents but also some results obtained in humans.

## Substrates vs. Modulators

Considerable work investigates the biological components of the substrate mechanisms of memory formation. These substrate mechanisms include changes in protein and gene expression and alterations in synaptic structure and function and are commonly considered the substrates of memory and neural plasticity (e.g., Kandel, 2001; Miyashita et al., 2008; Bekinschtein et al., 2010; Cheng et al., 2010; Roth et al., 2010; Johansen et al., 2011). These changes are initiated by a host of transient responses such as activation of transcription factors that regulate gene expression, activation of intracellular molecular signaling factors that regulate transcription factors, and alterations in calcium to regulate cell signaling factors. This list, especially if it were filled with specifics, would include serial and parallel processes that rival the central nervous system itself in complexity.

The cellular cascades constituting the substrates of neural plasticity can be initiated by the neurochemical signals that respond to an experience. While some of these cascades may be the brain’s memory of an experience *per se*, others are act to modulate downstream processes within the cellular machinery. We take the view that the processes that trigger and modulate mechanisms that produce long-lasting changes in the brain in response to experiences engage and amplify or diminish key neural responses to promote or impair memory formation. In this biological scheme, neuroendocrine responses to an experience modulate the formation of memory, augmenting the long-lasting impact an experience will have on brain function, with the neuroendocrine responses themselves dissipating soon after the event, though there may also be consequences of the hormonal responses too that long outlast the hormonal signal and experience.

**Figure 1:**
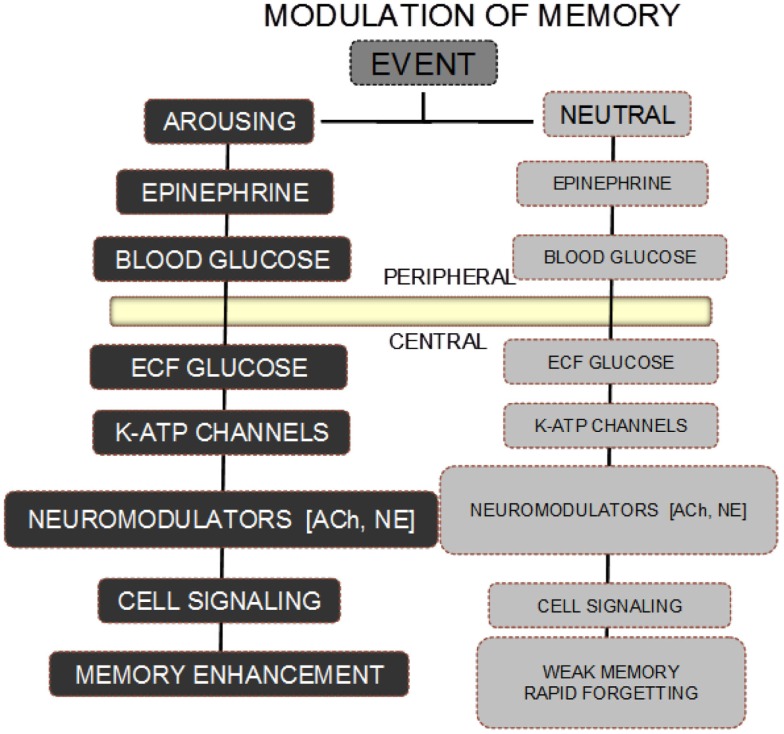
**Scheme for modulation of memory by epinephrine and glucose**. A neutral event fails to initiate the release of epinephrine, resulting in absence of the biological steps downstream from an increase in circulating epinephrine levels. As a result, the memory for a neutral event is weak and rapidly forgotten. In contrast, an arousing event results in increases in epinephrine released from the adrenal medulla. The epinephrine in turn initiates glycogen breakdown to glucose in the liver, with increases in blood glucose levels. Glucose enters the brain, providing support for neurotransmitter release, activation of intracellular signals responding to receptor binding, with eventual enhancement of memory.

The scheme shown in Figure 1 illustrates one overview of the different neurobiological consequences of an arousing vs. neutral event that may respectively be remembered well or quickly forgotten. In this scheme, an arousing event triggers the release of epinephrine with subsequent downstream actions that end with augmentation of neurochemical responses to the arousing event.

## Emotion and Arousal – Role of Epinephrine

Of hormonal modulators of memory, one of the earliest (Gold and van Buskirk, 1975) and perhaps best-studied is epinephrine (cf. Gold and McGaugh, 1975; McGaugh and Roozendaal, 2002; Korol and Gold, 2007). Epinephrine is released into blood from the adrenal medulla, with the magnitude of release graded across arousal conditions. For example, placement of a rat into a novel environment results in a twofold increase in circulating epinephrine levels. Epinephrine levels increase after foot shock, in an intensity-dependent manner resulting in a four- to 10-fold increase. A more stressful experience is immersion in a tub of water, as in the swim task (often called the Morris water maze), a condition in which epinephrine levels in blood can increase as much as 20 times above baseline (cf. Gold and McCarty, 1995).

When injected near the time of training, epinephrine enhances memory for learned information in rats (Gold and van Buskirk, 1975, 1978; Sternberg et al., 1985; Williams and Clayton, 2001; McGaugh and Roozendaal, 2002) as well as in humans (Cahill and Alkire, 2003). An early demonstration of memory enhancement by epinephrine was performed using rats trained in a widely used inhibitory avoidance task. This task uses a two-compartment alley in which a well-lit start compartment is separated from a dimly lit shock compartment. Upon crossing from the lit to dark compartment, which rats typically do to escape the unfavorable bright light, the rats receive a brief foot shock. During later memory testing, rats are placed back into the light compartment and evaluated for latency to cross into (or how long they avoid crossing into) the now safe shock compartment. In the absence of experimental intervention, it is unremarkable that the latency to avoid the shock compartment is a function of shock intensity: high intensity shocks are more aversive than are low intensity shocks and result in better avoidance of the shock compartment. Importantly, high intensity shock activates neuroendocrine responses that are substantially greater than responses to low intensity shock and that produce stronger and more lasting memory for the training experience (cf., Gold and McCarty, 1995; Gold and Korol, 2010).

**Figure 2:**
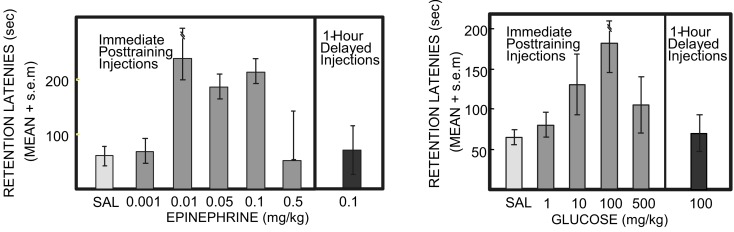
**Epinephrine and glucose enhancement of memory in rats**. Rats were trained in a one-trial inhibitory avoidance task, received injections of saline, epinephrine, or glucose immediately after training, and were tested 24 h later. Note the inverted-U dose-response curves for enhancement of memory seen on the test trial. Note also that injections of epinephrine or glucose 1 h after training did not significantly enhance memory on tests 24 h later. Under other conditions, e.g., training with a higher footshock, high doses of epinephrine, and glucose impair memory (Left from Gold and van Buskirk, 1975; Right from Gold, 1986).

If the neuroendocrine response serves as a measure for the emotional intensity of the experience itself, then it should be possible to create experimentally a more intense experience by administering the hormonal consequences of that intense experience. To test this, rats received an injection of epinephrine immediately after training with a low intensity shock. When memory was assessed the next day, those rats that received a post-training injection of epinephrine avoided the shock with longer latencies to re-enter the shock compartment, i.e., the rats avoided the low intensity shock as they would a higher intensity shock. The doses of epinephrine optimal for enhancing memory, as in Figure 2 (left), produce circulating epinephrine levels that mirror those seen in rats after a high intensity shock. Therefore, it appears that mimicking the physiology of an emotional event can result in better memory for that event, suggesting that hormonal responses to emotion can “tag” a memory, or more precisely a time, for events that are important.

Findings like these suggest that emotions can enhance memory by engaging neuroendocrine concomitants of the experiences (to regulate memory formation. However, the relationship between hormonal activation and memory formation is non-linear, following an inverted-U dose-response function, as in Figure 2. The inverted-U dose-response relationship, also termed hormesis, is seen across a wide range of cellular and organismic responses to many agents. Hormesis involves beneficial effects at low levels of a factor and impairing effects at high levels of the factor (Calabrese, 2008; Mattson, 2008). Here, it is the hormonal regulation of memory that follows the inverted-U curve. However, hormesis is also evident within memory research for other treatments, surprisingly including even β-amyloid peptides generally associated with Alzheimer’s Disease but which also enhance memory at low doses and impair memory at high doses (Morley and Farr, 2012; Puzzo et al., 2012).

With specific regard to memory, there are several interpretations possible for the upper end of the inverted-U where impairments occur, including ideas at different levels of analysis (cf. Gold, 2006; Calabrese, 2008; Mattson, 2008). At a cognitive level of analysis, it is possible that the impairments at the high end of the inverted-U relationship might reflect memory that is overly complete, with memory for extraneous information interfering with memory for the key information at times of retrieval. This view might be characterized as one in which learned information is embedded in too much “noise,” making it difficult to extract the relevant from irrelevant information on test trials. According to this view, the inverted-U is a result of a linear increase in memory to the point of interference with specific recall. A possible biological mechanism is that high levels of epinephrine might engage additional systems- or cellular-level biological mechanisms that impair memory, perhaps including overcompensation during a homeostasis response or activation of opiate mechanisms that serve as endogenous down-regulators of memory formation; in particular, amnesia produced by high epinephrine doses can be blocked by opiate antagonists (Izquierdo, 1982; Introini-Collison and McGaugh, 1987). In contrast to the cognitive interpretation of too much memory, this biological view suggests that the inverted-U represents two separate possibly linear processes, an ascending arm by which memory formation is facilitated intersecting with a descending arm reflecting diminishing enhancement or even memory impairment.

## Glucose as a Mediator of Epinephrine Effects on Memory

Epinephrine does not cross readily from blood to brain (Axelrod et al., 1959) and therefore requires a peripheral action to mediate its effects on brain mechanisms of memory. One peripheral intermediary between epinephrine and enhancement of memory is glucose. Glucose levels increase in blood quickly in response to circulating epinephrine, largely by initiating the formation of glucose from glycogen storage in the liver. Glucose, in turn, is taken from blood into the brain via active uptake mechanisms, where glucose acts directly on several brain sites to enhance memory formation.

Like epinephrine, peripherally administered glucose enhances memory in laboratory rodents on a wide variety of tasks (for reviews: White, 1991; Gold, 2001, 2008; Korol, 2002; Messier, 2004), like epinephrine with an inverted-U dose-response curve as in Figure 2 (right; e.g., Gold, 1986; Hall and Gold, 1986). The glucose doses that enhance memory result in blood glucose levels comparable to those seen after epinephrine doses that enhance memory. Moreover, when peripherally administered adrenergic receptor antagonists are used to block epinephrine effects on memory, the subsequent blood glucose levels, altered by blocking hepatic epinephrine receptors, again correspond to the drug effects on memory: very low and very high concentrations of blood glucose are found in conditions of poor memory while moderate levels correspond to good memory (Hall and Gold, 1986). To our knowledge, there are no explicit results that relate directly to mechanisms responsible for the falling phase of the dose-response curve.

If glucose delivery to the brain after epinephrine release mediates the effects on memory, then microinjections of glucose into specific brain regions should also enhance memory. Enhancement of memory with central injections of glucose have been seen in many circumstances, including after glucose infusions into the hippocampus, medial septum, amygdala, and striatum (e.g., Ragozzino et al., 1995, 1998; Parent and Gold, 1997; Parent et al., 1997; McNay and Gold, 1998; McNay et al., 2000; Stefani and Gold, 2001; Canal et al., 2005; Pych et al., 2006). Some evidence suggests that peripheral and central insulin levels also influence cognitive functions (e.g., Babri et al., 2007; Moosavi et al., 2007; cf. Craft, 2007; Craft et al., 2012), opening the possibility that some effects of glucose on memory may be secondary to insulin responses. While insulin may itself modulate memory processes, findings that direct brain microinjections of glucose influence memory, in a task × brain area specific manner, suggest that circulating insulin responses are not necessary for glucose to enhance memory, though CNS insulin may be involved (e.g., Zhao et al., 2004). Moreover, the issue of whether insulin crosses from blood to brain is not fully resolved highlighting the need to identify a proxy for insulin’s memory-modulating effects.

It may be surprising that glucose administration to the brain could enhance learning and memory given that it was once believed that brain extracellular glucose levels saturated uptake mechanisms in reasonably sated mammals. According to this view, additional glucose in blood or brain would be expected to have weak or no effect on neural functions. However, more recent information indicates that extracellular fluid (ECF) glucose levels in the brain are lower than previously thought. The principal source of glucose for the brain is from the blood in the cerebral vasculature (Siesjö, 1978), from where glucose crosses the blood-brain barrier via both facilitated and non-facilitated diffusion into the cerebrospinal fluid (CSF) and the ECF. Current estimates of glucose concentrations that saturation neuronal uptake of glucose are about 1.3 mM (Braun et al., 1985; Fellows et al., 1992). This value is close to the extracellular concentrations of glucose in the hippocampus of rats, ∼1.0 mM, as determined by direct measurements (Fellows et al., 1993; McNay and Gold, 1999). Also, NMR studies in humans give a very similar result for extracellular brain glucose levels (Gruetter et al., 1998). Thus, several lines of converging evidence demonstrate that basal extracellular glucose concentrations in the brains of both humans and rats are about 1 mM, and suggest that fluctuations in brain glucose levels and local use of glucose in different brain regions may be functionally important to optimal neural processing (McNay and Gold, 2002).

Considerable evidence indicates that extracellular glucose levels do in fact change during memory testing and that the changes are task- and region-specific. Extracellular concentrations of glucose in the hippocampus and striatum of rats were measured during performance of a spontaneous alternation task that assesses spatial working memory believed to tap hippocampus functions (McNay et al., 2000, 2001; Newman et al., 2011). Importantly, this task involves neither aversive nor appetitive rewards or stimuli, thus minimizing alterations in ECF glucose subsequent to changes in blood glucose that may occur with stress or food reward, for example. This task therefore provides information about glucose levels in the brain under non-emotional conditions of cognitive activity. In young adult rats, hippocampal ECF glucose concentrations decrease significantly during the behavioral testing period. Moreover, peripheral injections of glucose prior to behavioral testing enhance memory scores and block the testing-associated drop in ECF glucose in the hippocampus. Measures compared when varying task difficulty (3- vs. 4-arm mazes) showed that the decreases in ECF glucose levels varied with cognitive demands and not simply with locomotor activity. In addition, ECF glucose levels did not drop in the dorsal striatum, a brain area not implicated in processing memory in the spontaneous alternation task. The conclusion is that the neural activity required during memory testing consumes glucose in specific brain regions and that increases in circulating glucose levels fill the depletion resulting from this activity. In the spontaneous alternation task, the depletion is readily evident because the task is relatively free of stress and emotion. Under conditions of high emotion, epinephrine release into blood would initiate endogenous increases in blood glucose levels, thereby up-regulating memory, accomplishing endogenously what is produced experimentally in the example of the spontaneous alternation task.

Much is known about downstream cellular mechanisms that may contribute to glucose effects on memory. In particular, there is evidence that glucose effects on memory interact with several neurotransmitter systems to modulate memory. Evidence from many laboratories indicates that systemic glucose injections can reverse memory impairments produced by drugs that target several neurotransmitters, including glutamate, opiate, GABA, NE, and ACh (e.g., Gold, 1991; Stone et al., 1991; Walker and Gold, 1992; Ragozzino and Gold, 1995; Parent and Gold, 1997; Kopf et al., 1998, 2001; Pavone et al., 1998; Messier et al., 1999). The evidence is strongest for a role of ACh in contributing to the effects of glucose on memory, with many reports showing that glucose augments cholinergic functions (e.g., Messier et al., 1990, 1999; Durkin et al., 1992; Kopf and Baratti, 1994, 1995; Froelich et al., 1995; Micheau et al., 1995; Ragozzino et al., 1996, 1998; Kopf et al., 1998, 2001; Parkes and White, 2000). Of these, the most direct evidence comes from experiments showing that glucose augments acetylcholine release in the context of memory processing (e.g., Ragozzino et al., 1996, 1998; Messier et al., 1999). Acetylcholine, like some other neurotransmitters, has neuronal modulatory actions that amplify glutamate excitatory and GABA inhibitory mechanisms at neurophysiological and molecular levels of analysis (cf. Katz, 1999). It is this amplification of the impact of cell–cell communication during the time after an experience that may be one mechanism of the neurobiological basis for enhancement of memory by glucose.

In addition to interactions with neurotransmitter function, glucose may enhance memory through its action as an important substrate for energy production in the brain. However, glucose delivery to neurons is not always adequate to support optimal neural processing during conditions of high brain activation or low energy states. Astrocytic glial cells act as another energy source for neurons by providing lactate as an alternate energy substrate, thereby augmenting the energy derived from glucose uptake into neurons. Unlike neurons, astrocytes readily store glycogen that can be rapidly metabolized upon activation of glial neurotransmitter receptors to provide energy substrates such as lactate to neurons (Brown et al., 2004; Magistretti, 2006; Pellerin et al., 2007). Lactate, in turn, is taken into neurons and used as a substrate for energy metabolism. According to this view, basal levels of ECF glucose can fulfill neuronal energy requirements under low-need conditions. But when the need is greater, for example during more intense cognitive functions, astrocytic glycogenolysis is activated to provide lactate, which is transported to neurons to provide a rapid boost from glial energy reserves (Chuquet et al., 2010; Newman et al., 2011; Suzuki et al., 2011).

Within this framework, glucose can act by two routes – uptake into neurons to modulate memory or uptake into astrocytes to produce glycogen stores. The astrocytic glycogen would then be available to provide additional substrates following activation of glycogenolysis by cell–cell communication via glial receptors. Thus, astrocytes may be able to supplement glucose with lactate as a source of energy provisions to regulate processing at a cellular level and more broadly to modulate memory.

**Figure 3:**
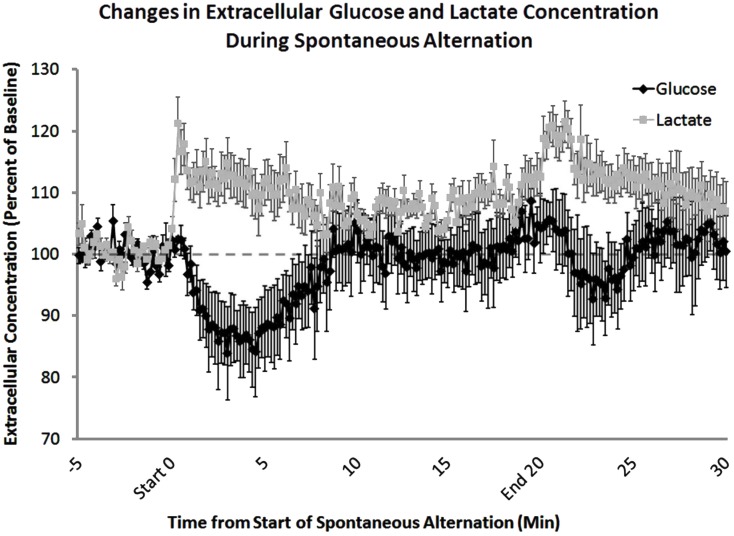
**Extracellular lactate and glucose levels in the hippocampus, measured before, during, and after behavioral testing**. Using lactate- and glucose-specific biosensors, extracellular concentrations of both lactate and glucose were measured during spontaneous alternation testing. Lactate concentrations increased significantly at the beginning of behavioral testing. In contrast, glucose concentrations decreased after 5 min on the task. The increase in extracellular glucose seen 5–10 min after the start of memory testing corresponds to an increase in blood glucose levels. After the rat was removed from the maze there was a significant increase in lactate compared to baseline levels most likely due to handling (From Newman et al., 2011).

A key role for glycogenolysis and lactate provision in regulating memory processing in the hippocampus was recently demonstrated through a series of experiments using *in vivo* assessment of extracellular lactate and glucose (Newman et al., 2011). Sensitive bioprobes were used to monitor, in 1 s measures, changes in extracellular levels of glucose and lactate in the hippocampus while rats were tested for working memory on a spontaneous alternation task (Figure 3). As seen using microdialysis methods, glucose levels decreased during testing. Of particular interest, however, is that lactate levels increased, mirroring over time the glucose responses. Close examination of the time courses of the reciprocal changes in glucose and lactate reveals that lactate apparently increases before the glucose drop. If the timing is confirmed, the likely scenario is that astrocytes are the target of neuro/glio/transmitters that initiate the breakdown of glycogen to produce and shuttle lactate to neurons. Of importance here, astrocytes have an abundance of receptors for many neurotransmitters, with several involved in initiating glycogenolysis. One of these is norepinephrine, which is released in brain in response to epinephrine, providing a very good bridge between emotions that promote memory processing, release of epinephrine peripherally, and norepinephrine centrally, and “on-demand” provision of energy substrates to facilitate the actions of neurons engaged in memory processing. Further evidence that the breakdown of glycogen to lactate is important for memory is that application of a drug to the hippocampus that blocks the breakdown of glycogen also impaired memory. The impairment was reversed by the addition by direct intra-hippocampal injection of glucose or lactate, showing that neurons could use either glucose or lactate to support memory functions, presumably using either to provide adequate metabolic substrates for neuronal mechanisms important for memory processes.

## Age-Related Memory Loss: Accompanied by Loss of Glucose Responses to Training

Rats and mice exhibit age-related impairments in learning and memory on many tasks. Often, the impairments can be characterized in terms of rapid forgetting, in which aged rats and mice have comparable learning and memory on tests soon after training, but poor memory at later times after training (Winocur, 1988; Barnes, 1991; Foster, 1999; Gold, 2001; Korol, 2002). There are many such examples of accelerated forgetting in aged rodents, with specific time courses that differ by task. Memory for inhibitory avoidance training, which remains stable for weeks after training in young rats, is intact soon after training and then deteriorates over the next several days (Gold et al., 1982). Rapid forgetting is also evident in the swim task, in which learning within a day appears to be forgotten overnight by aged but not young rats (Gage et al., 1984; Rapp et al., 1987; Mabry et al., 1995a). Similarly, young and aged rats have comparable memory scores on a reward reduction task when tested 1 day after training, but aged and not young rats exhibit forgetting when tested 7 days after training (Salinas and Gold, 2005). Other examples include more rapid forgetting in aged than young rats and mice on visual discriminated avoidance (Gold et al., 1982), spatial (Barnes and McNaughton, 1985), spatial reversal (Zornetzer et al., 1982), spontaneous alternation (Stone et al., 1992), and odor-reward association (Roman et al., 1996).

We have conducted a wide range of experiments to determine whether the modulators of memory, generated endogenously by training in young rats, were intact in aged rats and, if not, whether interventions might be effective at enhancing memory. The findings indicate that blood glucose responses to training or stress are severely attenuated in aged rats. For example, when aged rats are immersed in water as in the swim task, they exhibit only a minimal increase in blood glucose levels compared to that seen in young rats (Mabry et al., 1995a). Similarly, blood glucose levels increase in young adult rats as foot shock intensity, as in inhibitory avoidance training, is increased. However, aged rats do not (Mabry et al., 1995b). Interestingly, old rats do show an epinephrine response, which may actually be exaggerated compared to the response in younger counterparts. Thus, a key element important for providing the physiological consequence of emotion, i.e., epinephrine-induced release of glucose from the liver, is lost in the aged rats.

Revealing the importance of the absent glucose response to training, systemic injections of glucose enhance memory in aged rodents tested for inhibitory avoidance (Morris et al., 2010), reward reduction (Salinas and Gold, 2005), object recognition (Winocur and Gagnon, 1998), and spontaneous alternation (Stone et al., 1992; McNay and Gold, 2001), reversing age-related memory deficits on each of these tasks. In addition to results obtained with systemic administration of glucose, recent evidence shows that direct injections of glucose into the hippocampus restore memory in aged rats to the scores seen in young adult rats (Morris and Gold, 2012), supporting the theory that glucose acts directly in the brain to mediate its effects on memory. Studies using orally administered glucose in healthy young, aged, and cognitively impaired humans have shown complementary results, with glucose again having an inverted-U dose-response curve and enhancing memory on a range of tasks (cf. Korol, 2002; Messier, 2004; Gold, 2005). Some of the largest effects of glucose on memory in humans have been seen in healthy elderly people and in people with Alzheimer’s Disease, particularly for tasks that reveal memory impairments, with enhancement of memory for a narrative prose passage of 30–40% in healthy individuals and as much as 100% in Alzheimer’s patients (Manning et al., 1990, 1993; cf.: Gold, 2001; Korol, 2002).

Relating the findings in rats to mechanisms by which glucose enhances memory, release of acetylcholine, along with other neurotransmitters, is diminished in aged rats. Glucose enhancement of memory is accompanied by an increase in training-related release of acetylcholine in aged rats (Morris et al., 2010). Moreover, cellular responses to training subsequent to receptor activation on neurons are also diminished. One of these is activation of a transcription factor, CREB, after training. When enhancing memory, glucose also augments CREB activation (Morris and Gold, 2012).

The enhancement of memory by glucose in aged rodents, as well as in humans, is remarkably robust and returns memory formation and maintenance fully to levels seen in young adults. One implication of these findings is the aged brain *can* store and remember new memories as well as a younger brain but it does not do so because the modulatory systems that provide the biological bases of the significance of an experience are impaired. In this respect, the brain mechanisms of memory are not themselves impaired but have diminished levels of function because of poor peripheral responses to arousal. Thus, rapid age-related forgetting in old rats may reflect a primary physiological deficit of diminished ability to generate increases in blood glucose levels, i.e., an inability to engage the physiological sequelae through which emotions promote memory processing. In a sense, even seemingly salient events are non-emotional for aged rats and are not remembered well.

## Emotions Alter the Balance between Brain Memory Systems

Thus far, this review has focused on a physiological system that conveys significance of an experience to the brain, augmenting memory processes when doing so. However, when considering interactions across brain memory systems, the full story is far more complex than this. As discussed below, there is evidence for competition between memory systems for control of what is learned and used to guide behavior. Enhancement of one memory system can interfere with the function of another, resulting in a condition that simultaneously improves some types of memory and impairs other types of memory. This section will discuss how these results may apply to emotions and memory.

Findings first obtained with lesion experiments and later supported by other methods support the view that there are multiple memory systems in the mammalian brain (cf. Kim and Baxter, 2001; White and McDonald, 2002; Poldrack and Packard, 2003; Gold, 2004; Korol, 2004; Mizumori et al., 2004; Kesner, 2009), each with specialized roles in the formation of specific types of memory and used for different types of learning strategies. The evidence for this in rats includes triple dissociations for different classes of learning and memory, in which damage to one of three memory systems impairs memory for only one of three different tasks (e.g., Packard et al., 1989; Kesner et al., 1993; McDonald and White, 1993; Matthews et al., 1999). In particular, damage to the hippocampus impairs spatial (cognitive, place, win-stay) learning but does not alter egocentric (habit, response, win-shift) learning. Conversely, damage to the striatum impairs response but not place learning. In the context of multiple memory systems, damage to the amygdala impairs learning of highly emotional events, whether appetitive or aversive. Of note, the amygdala also participates more broadly in memory by modulating memories for experiences particularly sensitive to damage of other brain regions (McGaugh et al., 1996).

However, it is incomplete to say that learning in these tasks is based on a single memory system. Often lesions in one system enhance the learning of tasks associated with another system (Packard et al., 1989; McDonald and White, 1993; Matthews et al., 1999; Ferbinteanu and McDonald, 2001; Stone et al., 2005). These findings support the interpretation that activity in one neural system can interfere with behavioral output based on processing in another neural system. Moreover, pharmacological and hormonal manipulations of each memory system can alter the balance between memory systems, shifting the strategy a rat uses to solve a task (e.g., Packard and McGaugh, 1996; Packard, 1999; Conrad et al., 2001, 2004; Korol and Kolo, 2002; McIntyre et al., 2002, 2003a,b; Korol et al., 2004; McElroy and Korol, 2005; Zurkovsky et al., 2006, 2007).

A clear example of how the shift across systems can be modulated by hormones comes from studies of reproductive hormones. Across a range of experiments, estrogens have been shown to produce opposing effects on cognition, shifting the strategy used to solve a task. Bringing coherence to this field are demonstrations that estradiol treatments indeed did both, with tasks sorting according to the canonical neural system associated with the specific task to be learned. For example, under conditions of high levels of estrogens, rats show enhanced learning and memory of hippocampus-sensitive tasks, such as allocentric place learning, but impaired ability to learn striatum-sensitive tasks including those requiring stimulus-response or cued strategies (Korol and Kolo, 2002; Daniel and Lee, 2004; Korol et al., 2004; Davis et al., 2005; Zurkovsky et al., 2006, 2007, 2011).It is important to note that response learning is actually improved under low hormone states suggesting that estrogens shift the effective cognitive strategy and that in some contexts low hormonal states support better learning and memory.

Extensive evidence demonstrates that stress influences learning and memory, impairing or enhancing learning and memory under different conditions including estrogen status or whether tested in males or females (Conrad et al., 1996, 1999; Bowman et al., 2001; McEwen, 2001; Shors, 2001, 2006; Beck and Luine, 2002, 2010; Wright and Conrad, 2005; Diamond et al., 2007). Exposure to stressful stimuli that enhance trace eye blink conditioning in males, a task believed to depend on an intact hippocampus, disrupts learning, and memory in female rats. Replacement of estrogens to ovariectomized rats converts enhancements in learning by stress to impairments, suggesting that circulating ovarian hormones predisposes female rats to stress-related impairments for hippocampus-sensitive tasks. Whether or not the same sex or hormone by stress interactions would be seen for striatum-sensitive tasks is not currently known.

Of particular interest here is evidence that the balance between memory systems is modulated by stress and anxiety (Packard and Cahill, 2001; Packard, 2009). For example, stress near the time of training can shift rats toward the use of response solutions and away from the use of place solutions to solve learning tasks (Kim and Baxter, 2001; Sadowski et al., 2009), showing that stress, like estrogen status, can enhance, or impair learning depending on the task and the neural system tapped by that task. Specifically, stressors shift the preferred strategy expressed by rats from place (hippocampal) to response (striatal) solutions, with rats showing impaired learning for tasks that can be solved by a place strategy and enhanced learning for tasks that can be solved by response learning (Kim et al., 2001; Sadowski et al., 2009, Figure 4). Stress also leads to an increased use of stimulus-response/habit learning strategy vs. spatial learning strategy in humans (Schwabe et al., 2007, 2008; Dias-Ferreira et al., 2009).

**Figure 4:**
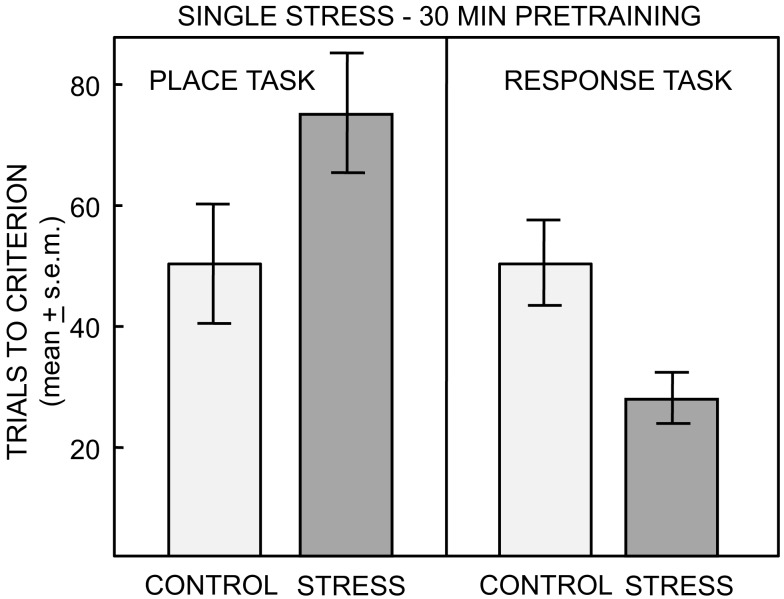
**Number of trials to reach criterion on the place and response task after no treatment or single restraint stress ending 30 min prior to training**. ANOVAS revealed a significant interaction of task by treatment. Pre-training single stress impaired learning on the place task and significantly enhanced learning on the response task (From Sadowski et al., 2009).

While the findings seem clear that stress induces changes in the balance across multiple memory systems, a specific neuroendocrine basis for these effects is less clear. Recent evidence shows that corticosteroids, like stress, promote a switch between memory systems in mice (Schwabe et al., 2012). The evidence linking epinephrine and glucose to altered participation of multiple memory systems is at present indirect. Release of acetylcholine in the striatum and hippocampus may contribute to the switch between memory systems (Gold, 2003); acetylcholine release in these systems is augmented by glucose administration during training (Ragozzino et al., 1996). Extracellular lactate levels increase in a task by brain area-dependent manner, suggesting that astrocytic glycogen breakdown to lactate may also contribute to the functions of multiple memory systems as might release of other signaling molecules such as neurotrophic factors (Scavuzzo et al., 2011; Korol et al., 2012). Explicit tests of the relationships between these measures and stress effects on multiple memory systems remain to be performed.

## Conclusion

Research on modulation of memory has revealed two important ways in which emotion, not distinguished here from arousal, can influence memory. The first is that physiological concomitants of emotion modulate memory. At a physiological level, emotional level, and memory are related in an inverted-U manner. Moderate arousal enhances memory and very high arousal impairs memory. In this way, emotions can be either good or bad factors for memory processing. The bases for these relationships appear to be found through a biology that cross many systems, in particular the adrenal gland, liver, blood, and brain. It is worth noting that there is now extensive research on humans confirming the main effects of glucose on memory (Gold, 2001; Messier, 2004; Smith et al., 2011), although differences across species are very likely to emerge with further research.

The up- and down-regulation of memory processing by physiological responses to emotion also has another dimension in shifting the strategy used to solve a problem. Evidence in rats suggests that high emotion shifts rats away from place learning strategies and toward response learning strategies. In terms of associated neural systems, the shift appears to be from hippocampus to striatum control over learning strategy.

The intersection of these modes of emotional effects on memory makes the relationships complicated but certainly tractable. Far more attention is needed to identify the conditions and mechanisms through which the convergence of the neuroendocrine responses to emotion with enhancement and impairment of memory is expressed.

## Conflict of Interest Statement

The authors declare that the research was conducted in the absence of any commercial or financial relationships that could be construed as a potential conflict of interest.
